# Pancreatic β-Cell Dysfunction Is Associated with Nonalcoholic Fatty Liver Disease

**DOI:** 10.3390/nu13093139

**Published:** 2021-09-09

**Authors:** Xu Chen, Jinghe Xiao, Juan Pang, Shen Chen, Qing Wang, Wenhua Ling

**Affiliations:** 1 Department of Nutrition, School of Public Health, Ningxia Medical University, Yinchuan 750004, China; chenx93@mail.sysu.edu.cn; 2 Department of Nutrition, School of Public Health, Sun Yat-sen University, Guangzhou 510080, China; 13192696141@163.com (J.X.); pangj7@mail2.sysu.edu.cn (J.P.); 3 Department of Toxicology, School of Public Health, Sun Yat-Sen University, Guangzhou 510080, China; chensh325@mail.sysu.edu.cn (S.C.); wangq27@mail.sysu.edu.cn (Q.W.)

**Keywords:** NAFLD, pancreatic function, HOMA, NHANES III, beta cell compensation

## Abstract

Background: Nonalcoholic fatty liver disease (NAFLD) is associated with decreased insulin sensitivity. However, the association between NAFLD and pancreatic β-cell function is still ambiguous. Here, we assessed whether pancreatic β-cell function is associated with NAFLD. Method: The data of NHANES III from 1988 to 1994 were used. NAFLD was diagnosed when subjects had ultrasonographically hepatic steatosis without other liver diseases. Disposition index (DI) was employed to assess pancreatic β-cell function. A total of 6168 participants were included in this study. Results: NAFLD participants had much higher HOMA2-%B (weighted mean, 124.1; standard error, 1.8) than the non-NAFLD participants (weighted mean, 100.7; standard error, 0.9). However, when evaluating the β-cell function in the context of insulin resistance by using DI index, DI levels were much lower in NAFLD subjects (weighted mean, 79.5; standard error, 1.0) compared to non-NAFLD (weighted mean, 95.0; standard error, 0.8). Multivariate logistic regression analyses showed that DI was inversely associated with NAFLD prevalence. The adjusted OR (95% CI) for quartile 1 versus quartile 4 was 1.81 (1.31–2.50) (*p* < 0.001 for trend). Moreover, DI was also inversely associated with the presence of moderate to severe hepatic steatosis. The multivariable-adjusted ORs across quartiles of DI were 2.47, 1.44, 0.96 and 1.00 for the presence of moderate to severe hepatic steatosis (*p* < 0.001 for trend). Conclusions: Pancreatic β-cell function might be a new predictor for the presence of NAFLD, and insufficient compensatory β-cell function is associated with NAFLD.

## 1. Introduction

With the development of a social economy and changes of lifestyle, the prevalence of noncommunicable chronic disease has become a huge burden all over the world. Nonalcoholic fatty liver disease (NAFLD) is regarded as the manifestation of metabolic syndrome in the liver [1]. The global prevalence of NAFLD was about 24% in 2016 [2]. NAFLD prevalence is higher in developed countries, and one-third of the US population are affected by NAFLD [3]. NAFLD disease encompasses a spectrum from simple steatosis to non-alcoholic steatohepatitis (NASH), which can lead to cirrhosis and hepatocellular carcinoma. Furthermore, NAFLD is a multiorgan disease with extrahepatic manifestations such as type 2 diabetes, atherosclerosis and kidney disease [4]. Therefore, it is necessary to better understand the mechanisms and predictors of NAFLD to influence future disease treatment and screening.

Reduced insulin sensitivity and pancreatic β-cell dysfunction interact to promote type 2 diabetes development. Without β-cell dysfunction, reduced insulin sensitivity alone will not progress to diabetes [5]. NAFLD is characterized by insulin resistance in the liver and NAFLD also increases the risk of type 2 diabetes [6]. However, evidence of the association between NAFLD and β-cell function is limited.

The Homeostasis Model Assessment (HOMA) evaluates pancreatic β-cell function (%B) and body insulin sensitivity (%S) as percentages of a normal reference population [7]. HOMA-%B is widely used for estimating β-cell function in large-scale studies. However, the β-cell function is known to be compensatorily enhanced by ambient insulin resistance [8]. The disposition index (DI) has presented a valuable tool for the assessment of β-cell function, which distinguishes the insufficient compensation of β-cell function in the context of insulin resistance [9,10]. Therefore, we explored the association of β-cell function with NAFLD by using HOMA2-%B and DI indices in this study.

## 2. Methods

### 2.1. Study Participants

This study was conducted using data from the 3rd National Health and Nutrition Examination Survey (NHANES III), which was conducted to evaluate the health and nutritional status of individuals in the United States. The institutional review board of the National Center for Health Statistics approved this survey study and informed consent forms had been signed by all participants. First, participants who were >20 years old and received laboratory examination were included (*n* = 18,825). Then we excluded the subjects with missing data on fasting plasma glucose levels and serum C-peptide, and missing hepatic ultrasonography data (*n* = 5575). In addition, subjects with heavy alcohol drinking habits (>average 3 drinks per day in men or >average 2 drinks per day in women), diabetes (a self-reported diagnosis or treatment with hypoglycemic medications or fasting plasma glucose level ≥ 7.0 mmol/L or the HbA_1_c level ≥ 6.5%), iron overload (transferring saturation > 50%), viral hepatitis (positive in antibody of hepatitis C or surface antigen of hepatitis B examination), fasting time less than 8 h and pregnant women were also excluded (*n* = 6782). Finally, after excluding the participants with missing data on body mass index, serum aminotransferase, waist circumference and total cholesterol (TC) (*n* = 296), a total of 6168 subjects were included in the analysis.

### 2.2. Clinical Evaluation

Participants were defined as having hypertension if they received treatment with antihypertensive medication or had mean systolic blood pressure greater than 140 mm Hg or mean diastolic blood pressure greater than 90 mm Hg. Poverty was defined as a poverty income ratio <1.00. We categorized smoking status as current smokers, ex-smokers and never smokers. Individuals who had smoked at least 100 cigarettes during their lifetime and quitted smoking were treated as ex-smokers and current smokers were on-going smokers. Sedentary lifestyle was classified if participants answered “no” to all questions about engagement in exercise activities over the last month.

### 2.3. Definition of NAFLD

Three board-certified radiologists assessed the ultrasound images obtained in NHANES III to evaluate hepatic steatosis. The following 5 criteria were recorded: (1) the definition of the gallbladder walls, (2) liver-to-kidney contrast, (3) deep beam attenuation, (4) the brightness of the liver parenchyma, (5) echogenic walls in the small intrahepatic vessels. Finally, based on the above five parameters, the liver was categorized as normal to severe hepatic steatosis. In this study, NAFLD was defined as mild to severe hepatic steatosis without other liver diseases [11].

### 2.4. β-Cell Function Assessment

The HOMA2-%B was used to estimate insulin secretion. HOMA2-%S was used to estimate the insulin sensitivity. HOMA2-%B and HOMA2-%S were obtained using the online calculator (http://www.dtu.ox.ac.uk/homacalculator) by fasting plasma glucose levels and C-peptide levels (accessed on 20 March 2021) [12]. DI was calculated as HOMA2-%S * HOMA2-%B.

### 2.5. Statistical Analysis

According to the NHANES analytic guidelines, appropriate sampling weights, strata and primary sampling units were applied in the analyses to account for the oversampling of a certain subpopulation, unequal probability of selection and nonresponse adjustment. Linear regression was performed to compare the means of characteristics for continuous variables. Logistic regression was used to compare the proportions of characteristics for the categorical variables. Multivariate logistic regression models were performed to explore an independent association between pancreatic β-cell function and NAFLD after adjusting for potential confounders. Moreover, stratified analyses and interaction analyses were performed to examine whether the association differed by glycemia state, BMI, age and sex. All statistical analyses were carried out using complex sample modules of SPSS software, version 22.0 (SPSS, Inc., Chicago, IL, USA).

## 3. Results

### 3.1. Characteristics of Participants

In this study, 6168 NHANES survey subjects were included and 1970 participants had NAFLD. As presented in Table 1, compared with non-NAFLD subjects, NAFLD subjects were noted to be hypertensive, older, and have a sedentary lifestyle. NAFLD individuals were more likely to have larger waist circumference, higher BMI, and higher plasma levels of TC, AST, ALT and HbA1c. In addition, as for the β-cell function assessment, NAFLD subjects had higher HOMA2-%B levels but lower DI levels compared with non-NAFLD participants.

### 3.2. HOMA2-%B and NAFLD

To explore the association of HOMA2-%B with NAFLD, weighted logistic regression models were performed to assess the HOMA2-%B quartiles with NAFLD presence after adjusting for confounding variables in these models. The presence of NAFLD was treated as a binary outcome. As presented in Table 2, after adjusting for ethnicity, sex and age, higher HOMA2-%B quartiles were associated with higher odds of NAFLD (OR for the highest quartile versus the lowest quartile, 4.00; 95% CI, 3.22–4.96; *p*  < 0.001 for trend). This association was still significant after further adjusting for confounding variables in models 2 and 3. Moreover, we further investigated the association of HOMA2-%B with the presence of moderate to severe hepatic steatosis. As shown in Table 3, the fully adjusted ORs in model 3 across quartiles of HOMA2-%B were 1.00, 1.98, 2.91 and 3.74 for the presence of moderate to severe hepatic steatosis, respectively.

### 3.3. DI and NAFLD

β-cell function is known to be compensatorily enhanced by ambient insulin resistance. As shown above, we also found that HOMA2-%B was positively associated with the presence of NAFLD. In the context of insulin resistance, DI was used to assess whether the compensation of β-cell function was sufficient. Therefore, we next explored the association of DI with the presence of NAFLD. Interestingly, in contrast to HOMA2-%B, DI was found to be negatively associated with NAFLD (Table 4). After adjusting for confounding variables in model 1, the ORs across quartiles of DI were 3.64, 1.76, 1.04 and 1.00 for the presence of NAFLD (*p* for trend < 0.001), respectively. This association was still significant after further adjusting for confounding variables in models 2 and 3. In the fully adjusted model 3, the OR for the lowest quartile versus the highest quartile was 1.81 (CI, 1.31–2.50; *p*  < 0.001 for trend).

In addition, we also explored the association of DI with the presence of moderate to severe hepatic steatosis. The results are shown in Table 5. Subjects with lower DI categories were associated with moderate to severe hepatic steatosis. In the adjustment for confounding variables in model 1, the OR for the lowest quartile versus the highest quartile was 7.88 (CI, 5.82–10.67; *p*  < 0.001 for trend), and the fully adjusted ORs in model 3 across quartiles of DI were 2.47, 1.44, 0.96 and 1.00 (*p*  < 0.001 for trend), respectively.

### 3.4. Subgroup Analyses

Effects of the interaction between baseline DI levels and glycemia state, BMI, age and sex were also examined. As shown in Table 6, the association between DI and the presence of NAFLD was evident in participants with BMI ≥ 25, but not in those with BMI < 25 (*p* for interaction < 0.05). There was no significant association between DI levels and the presence of NAFLD based on glycemia state, age and sex.

## 4. Discussion

The results of this study illustrate an association between pancreatic β-cell function and NAFLD. Firstly, we found that compared to non-NAFLD participants, NAFLD participants had much higher HOMA2-%B. However, when evaluating the β-cell function in the context of insulin resistance by using the DI index, we found that, interestingly, DI levels were much lower in NAFLD subjects than non-NAFLD subjects, and after adjusting for the potential confounders, an independent inverse association between DI and NAFLD was determined.

Insulin resistance and pancreatic β-cell dysfunction interact to promote type 2 diabetes development. Studies have demonstrated that even within a normal range of fasting plasma glucose, the β-cell function may begin to decline before the diagnosis of overt type 2 diabetes [13,14]. NAFLD is associated with decreased insulin sensitivity [15], and cohort studies showed that fatty liver increased the incidence of diabetes [16,17]. However, the evidence of the association between pancreatic β-cell function and NAFLD is limited and controversial. One study in Italy demonstrated β-cell function is lowered in biopsy-proven NASH patients, but not in simple steatosis subjects [18]. Norimasa et al. found that in NAFLD patients, HOMA-B% was decreased with the severity of liver fibrosis [19]. However, another study in the US found that HOMA-B% was remarkably higher in NAFLD participants and in those with β-cell dysfunction, even in simple steatosis patients [20]. The apparent controversy in these studies was due to several factors. Firstly, the genetical or racial difference in these study cohorts may partly explain the discrepancy. In addition, the sample size of these studies is small, which can lead to bias. More importantly, these studies selected specific stages of NAFLD. Here, we explored the relationship between NAFLD and β-cell function in the large US national population using NHANES III data. Our results suggest that the presence of NAFLD is associated with an enhanced pancreatic β-cell response, as evident by the increased HOMA2-B%. However, the increased β-cell response is insufficient in the context of insulin resistance, and we found DI was inversely associated with the presence of NAFLD. Overall, these results indicate that HOMA-2B% and DI might be new predictors for NAFLD, and establish the association between insufficient compensatory β-cell function and NAFLD.

Two factors may explain the association between pancreatic β-cell function and NAFLD. First, pancreatic β-cell dysfunction participates in the pathogenesis of glucose metabolism dysregulation and hyperinsulinemia, which may lead to hepatic lipid metabolism disorders and NAFLD progression [21]. Second, NAFLD is a multisystem disease [22], which may influence pancreatic β-cell function. The liver is able to secrete a lot of metabolites, proteins and extracellular vesicles, which play important roles in metabolic processes, both in the liver and in other tissues [23]. One study found that lipid-overload hepatocyte could promote endothelial inflammation and atherogenesis via extracellular vesicles [24]. Moreover, hepatokines, such as FGF21, which is secreted by the liver, exert powerful effects on pancreatic β-cell function [25,26]. However, it is worth noting that the detailed mechanism of the association between β-cell dysfunction and NAFLD is still unclear. More studies are needed to better clarify the mechanism.

DI and HOMA2-B% were employed to assess pancreatic β-cell function in this study. Compared with HOMA2-B%, DI is a better measurement of pancreatic β-cell function because it compensates for the ambient insulin sensitivity level [10]. Louise et al. also found that DI is more predictive for incident type 2 diabetes than HOMA2-B% and insulin resistance indices [5]. On the other hand, we calculated DI based on the HOMA2 model because the calculation of HOMA2 indices takes account of some physiological adjustments based on HOMA1. One Korean study showed that HOMA2 had significantly better discriminatory abilities than HOMA1 for the prediction of incident diabetes in non-diabetic subjects [27]. Moreover, we used fasting serum C-peptide data rather than insulin data to calculate β-cell function. C-peptide is produced in equal amounts as insulin, and the measurement of fasting serum C-peptide level can better reflect the insulin secretion ability of pancreatic β-cells because C-peptide is not degraded by the liver, and its half-life is significantly longer than that of insulin [28]. One study also showed that HOMA2-IR calculated with C-peptide was a more sensitive indicator for metabolic syndrome than HOMA2-IR calculated with insulin [29]. Above all, DI calculated with C-peptide is an easy-to-use, low-cost and relatively accurate indicator of pancreatic β-cell function, and can be used in large epidemiological studies. However, it is worth noting that DI is an estimate that only takes into account fasting glucose and C-peptide levels, which are relatively less predictive of β-cell function compared to other indices derived from the glucose challenge test [30].

There were several limitations in this study. First, hepatic ultrasonography was used to diagnose NAFLD. Hepatic ultrasonography is low-cost and is commonly used in epidemiological studies [31]. However, ultrasonography examination is not the gold standard for liver biopsy to diagnose NAFLD, and is unable to identify fatty infiltration below 30% [32]. Second, we assessed the β-cell function of DI based on the HOMA2 model, which was simple and easy-to-use, but less accurate in reflecting β-cell function compared to other indices derived from the glucose challenge test and hyperglycemic clamp method (the gold standard) [33]. Finally, the association between β-cell function and NAFLD was cross-sectional, which precluded us from ascertaining temporal associations and establishing causal relationship. Future cohort studies are required to determine the causal relationship between β-cell function and the presence of NAFLD.

## 5. Conclusions

To conclude, an inverse association between DI and the presence of NAFLD was found in this study. Our results suggest that pancreatic β-cell function might be a new predictor for the presence of NAFLD, and could establish the association between insufficient compensatory β-cell function and NAFLD.

## Figures and Tables

**Table 1:** Baseline characteristics by NAFLD status.

	NAFLD Status		
Variables	Non-NAFLD (*n* = 4198)	NAFLD (*n* = 1970)	*p* Value
Age (years)	40.0 ± 0.5	43.2 ± 0.6	<0.001
Gender, male (%)	47.0 (44.7–49.3)	51.1 (47.8–54.5)	0.083
Body mass index (kg/m^2^)	25.4 ± 0.1	28.4 ± 0.2	<0.001
Waist circumference (cm)	88.2 ± 0.3	96.2 ± 0.6	<0.001
Hypertension (%)	16.0 (14.1–18.1)	25.4 (22.8–28.3)	<0.001
Ethnicity (%)			<0.001
Non-Hispanic white	76.2 (72.9–79.1)	75.4 (71.4–79.1)	
Non-Hispanic black	11.3 (9.8–12.9)	8.4 (7.1–9.8)	
Mexican-American	5.0 (4.1–6.0)	6.7 (5.3–8.4)	
Others	7.6 (5.6–10.3)	9.5 (6.9–13.0)	
Smoking (%)			0.053
Never	47.6 (44.8–50.4)	46.2 (43.1–49.3)	
Ex-smoker	23.3 (21.4–25.3)	28.0 (24.8–31.3)	
Current smoker	29.1 (26.7–31.6)	25.9 (23.1–28.9)	
Poverty status (%)	11.4 (9.5–13.7)	11.1 (9.0–13.6)	0.791
Total cholesterol (mg/dL)	200.2 ± 1.0	204.2 ± 1.8	0.021
HDL-cholesterol (mg/dL)	51.3 ± 0.5	47.0 ± 0.7	<0.001
Hemoglobin A1c (%)	5.15 ± 0.02	5.23 ± 0.02	<0.001
Aspartate aminotransferase (IU/L)	19.6 ± 0.2	22.5 ± 0.3	<0.001
Alanine aminotransferase (IU/L)	15.2 ± 0.3	21.2 ± 0.7	<0.001
AST:ALT ratio	1.49 ± 0.03	1.28 ± 0.03	<0.001
Sedentary lifestyle (%)	11.1 (9.3–13.1)	14.5 (12.0–17.4)	0.006
HOMA2-%B	100.7 ± 0.9	124.1 ± 1.8	<0.001
Disposition index	95.0 ± 0.8	79.5 ± 1.0	<0.001

Data are presented as the weighted mean ± SEs or frequency (95% confidence intervals) as appropriate. NAFLD, nonalcoholic fatty liver disease; HDL, high density lipoprotein; AST:ALT, aspartate aminotransferase: alanine aminotransferase levels; HOMA2, homeostasis model assessment version 2.

**Table 2:** Adjusted odds ratios (95% confidence intervals) of NAFLD according to HOMA2-B% quartiles using logistic regression.

	HOMA2-%B				*p* for Trend
	Quartile 1 <80.1	Quartile 2 80.1–105.7	Quartile 3 105.7–135.1	Quartile 4 ≥135.1	
*n* = 6168	1540	1544	1541	1543	
Model 1	1.00	1.54 (1.18–2.00)	2.38 (1.73–3.29)	4.00 (3.22–4.96)	<0.001
Model 2	1.00	1.33 (0.99–1.79)	1.80 (1.27–2.55)	2.40 (1.84–3.14)	<0.001
Model 3	1.00	1.30 (0.96–1.76)	1.68 (1.19–2.38)	2.12 (1.61–2.80)	<0.001

Model 1: Ethnicity, sex and age were adjusted. Model 2: Variables in Model 1 plus sedentary lifestyle, hypertension, poverty status, smoking status, body mass index and waist circumference were adjusted. Model 3: Variables in Model 2 plus total cholesterol levels and alanine aminotransferase were adjusted.

**Table 3:** Adjusted odds ratios (95% confidence intervals) of presence of moderate to severe hepatic steatosis according to HOMA2-B% quartiles using logistic regression.

	HOMA2-%B				*p* for Trend
	Quartile 1 <80.1	Quartile 2 80.1–105.7	Quartile 3 105.7–135.1	Quartile 4 ≥135.1	
*n* = 6168	1540	1544	1541	1543	
Model 1	1.00	2.58 (1.67–3.99)	5.12 (3.36–7.79)	10.12 (7.13–14.35)	<0.001
Model 2	1.00	2.03 (1.28–3.21)	3.18 (2.00–5.05)	4.44 (2.90–6.79)	<0.001
Model 3	1.00	1.98 (1.22–3.23)	2.91 (1.81–4.67)	3.74 (2.41–5.83)	<0.001

Model 1: Variables including ethnicity, sex and age were adjusted. Model 2: Variables in Model 1 plus sedentary lifestyle, hypertension, poverty status, smoking status, body mass index and waist circumference were adjusted. Model 3: Variables in Model 2 plus total cholesterol levels and alanine aminotransferase were adjusted.

**Table 4:** Adjusted odds ratios (95% confidence intervals) of NAFLD according to disposition index quartiles using logistic regression.

	Disposition Index				*p* for Trend
	Quartile 1 <64.1	Quartile 2 64.1–82.2	Quartile 3 82.2–104.5	Quartile 4 ≥104.5	
*n* = 6168	1549	1534	1542	1543	
Model 1	3.64 (2.83–4.70)	1.76 (1.41–2.19)	1.04 (0.81–1.33)	1.00	<0.001
Model 2	2.06 (1.54–2.77)	1.23 (0.97–1.55)	0.86 (0.68–1.08)	1.00	<0.001
Model 3	1.81 (1.31–2.50)	1.16 (0.92–1.46)	0.80 (0.64–1.01)	1.00	<0.001

Model 1: Ethnicity, sex and age were adjusted. Model 2: Variables in Model 1 plus sedentary lifestyle, hypertension, poverty status, smoking status, body mass index and waist circumference were adjusted. Model 3: Variables in Model 2 plus total cholesterol levels and alanine aminotransferase were adjusted.

**Table 5:** Adjusted odds ratios (95% confidence intervals) of presence of moderate to severe hepatic steatosis according to disposition index quartiles using logistic regression.

	Disposition Index				*p* for Trend
	Quartile 1 <64.1	Quartile 2 64.1–82.2	Quartile 3 82.2–104.5	Quartile 4 ≥104.5	
*n* = 6168	1549	1534	1542	1543	
Model 1	7.88 (5.82–10.67)	3.00 (2.14–4.20)	1.53 (1.07–2.17)	1.00	<0.001
Model 2	3.10 (2.17–4.42)	1.64 (1.12–2.40)	1.09 (0.75–1.60)	1.00	<0.001
Model 3	2.47 (1.71–3.56)	1.44 (0.98–2.12)	0.96 (0.65–1.42)	1.00	<0.001

Model 1: Ethnicity, sex and age were adjusted. Model 2: Variables in Model 1 plus sedentary lifestyle, hypertension, poverty status, smoking status, body mass index and waist circumference were adjusted. Model 3: Variables in Model 2 plus total cholesterol levels and alanine aminotransferase were adjusted.

**Table 6:** Adjusted odds ratios (95% confidence intervals) of NAFLD according to DI quartiles using logistic regression in various subpopulations.

	Disposition Index				*p* for Trend	*p* for Interaction
	Quartile 1	Quartile 2	Quartile 3	Quartile 4		
Subpopulation						
Glycemia groups						0.152
Normal glycemia	1.72 (1.30–2.27)	1.09 (0.77–1.55)	1.01 (0.70–1.46)	1.00	<0.001	
Prediabetes	1.87 (1.18–2.98)	1.08 (0.60–1.92)	1.39 (0.78–2.49)	1.00	0.010	
BMI, kg/m^2^						0.009
<25	1.53 (0.97–2.39)	1.05 (0.65–1.70)	1.13 (0.69–1.84)	1.00	0.136	
≥25	2.44 (1.54–3.89)	1.93 (1.35–2.75)	1.45 (1.00–2.09)	1.00	<0.001	
Age groups, years						0.181
≤60	1.80 (1.30–2.51)	1.09 (0.88–1.36)	0.83 (0.64–1.06)	1.00	<0.001	
>60	2.10 (1.28–3.44)	1.57 (0.96–2.58)	1.15 (0.64–2.08)	1.00	0.003	
Sex						0.889
Male	1.88 (1.12–3.13)	1.28 (0.90–1.83)	0.77 (0.52–1.16)	1.00	0.006	
Female	1.86 (1.25–2.76)	1.16 (0.84–1.60)	1.04 (0.76–1.43)	1.00	0.005	

Variables including ethnicity, sex, age, body mass index, waist circumference, smoking status, poverty status, hypertension, sedentary lifestyle, serum alanine aminotransferase and total cholesterol levels were adjusted.

## Data Availability

Publicly available datasets were analyzed in this study. NHANES data can be found here: https://wwwn.cdc.gov/nchs/nhanes/Default.aspx (accessed on 1 March 2021).

## Abbreviations

ALT, alanine aminotransferase; BMI, body mass index; DI, disposition index; HOMA, the Homeostasis Model Assessment; NAFLD, nonalcoholic fatty liver disease; NASH, nonalcoholic steatohepatitis; NHANES, the National Health and Nutrition Examination Survey; OR, odds ratio.
